# Our Evolving Understanding of ME/CFS

**DOI:** 10.3390/medicina57030200

**Published:** 2021-02-26

**Authors:** Kenneth J. Friedman, Modra Murovska, Derek F. H. Pheby, Paweł Zalewski

**Affiliations:** 1 Department of Pharmacology and Physiology, New Jersey Medical School, Newark, NJ 07103, USA; 2 Institute of Microbiology and Virology, Riga Stradiņš University, LV-1067 Riga, Latvia; modra@latnet.lv; 3 Society and Health, Buckinghamshire New University, High Wycombe HP11 2JZ, UK; derekpheby@btinternet.com; 4 Department of Hygiene, Epidemiology, Ergonomy and Postgraduate Education, Ludwik Rydygier Collegium Medicum in Bydgoszcz Nicolaus Copernicus University in Torun, M. Sklodowskiej-Curie 9, 85-094 Bydgoszcz, Poland; p.zalewski@cm.umk.pl

**Keywords:** ME/CFS, Longhaul COVID-19, pathophysiology

## Abstract

The potential benefits of the scientific insights gleaned from years of treating ME/CFS for the emerging symptoms of COVID-19, and in particular Longhaul- or Longhauler-COVID-19 are discussed in this opinion article. Longhaul COVID-19 is the current name being given to the long-term sequelae (symptoms lasting beyond 6 weeks) of SARS-CoV-2 infection. Multiple case definitions for ME/CFS exist, but post-exertional malaise (PEM) is currently emerging as the ‘hallmark’ symptom. The inability to identify a unique trigger of ME/CFS, as well as the inability to identify a specific, diagnostic laboratory test, led many physicians to conclude that the illness was psychosomatic or non-existent. However, recent research in the US and the UK, championed by patient organizations and their use of the internet and social media, suggest underlying pathophysiologies, e.g., oxidative stress and mitochondrial dysfunction. The similarity and overlap of ME/CFS and Longhaul COVID-19 symptoms suggest to us similar pathological processes. We put forward a unifying hypothesis that explains the precipitating events such as viral triggers and other documented exposures: For their overlap in symptoms, ME/CFS and Longhaul COVID-19 should be described as Post Active Phase of Infection Syndromes (PAPIS). We further propose that the underlying biochemical pathways and pathophysiological processes of similar symptoms are similar regardless of the initiating trigger. Exploration of the biochemical pathways and pathophysiological processes should yield effective therapies for these conditions and others that may exhibit these symptoms. ME/CFS patients have suffered far too long. Longhaul COVD-19 patients should not be subject to a similar fate. We caution that failure to meet the now combined challenges of ME/CFS and Longhaul COVID-19 will impose serious socioeconomic as well as clinical consequences for patients, the families of patients, and society as a whole.

The development of the COVID-19 pandemic in 2020, caused by a high rate of human infection to the severe acute respiratory syndrome coronavirus 2 (SARS-CoV-2), and the unanticipated, subsequent, long-duration symptoms currently known as Longhaul COVID-19, challenges our past and present conceptualizations of ME/CFS: tens of millions of people have been infected with a specific, heretofore unknown virus [1], which has left thousands of patients chronically ill [2] with a set of symptoms remarkably similar to ME/CFS [3]. It is anticipated and estimated that approximately 10 percent of COVID-19 patients will develop Longhaul COVID-19 symptoms [4]. The occurrence of these symptoms subsequent to the acute phase of infection leaves little doubt as to the causation or that these symptoms represent a physiological abnormality.

Those of us who have been studying and researching post-viral syndromes for decades have no doubt that, as with post-viral syndromes following other viral infections, this displays the constellation of symptoms that come within the scope of myalgic encephalomyelitis/chronic fatigue syndrome (ME/CFS). ME/CFS has been a cause of considerable morbidity for a large number of patients for many years, but many have suffered from disbelief and lack of understanding on the part of doctors, and those doctors and researchers who appreciated the reality of the condition have often faced an uphill struggle to advance knowledge in this area. However, the scientific knowledge that has been acquired as a result of these endeavors can now serve the interests of the wider world community, which is experiencing at first hand the trauma of a post-viral syndrome. Moreover, intensive investigation of the causation and effective treatment of these symptoms now will result in improved understanding and treatment of these symptoms regardless of triggering illness.

Arriving at a case definition and understanding of ME/CFS has been, and continues to be, difficult. For decades, attempts to define and name the disease have transpired in parallel in the United States (US) and the United Kingdom (UK) [5]. In the UK, patients suffering acute illness, both in cluster outbreaks and sporadic occurrences, developed a pattern of chronic symptoms suggestive of myalgic encephalomyelitis. In the US, several cluster outbreaks of acute disease progressing to chronic disease with a similar portfolio of symptoms were identified. The majority of cases identified were sporadic (or isolated) cases leading to confusion as to the cause of the symptoms. This led to several descriptive characterizations: Yuppie Flu, Chronic Epstein Barr, Chronic Fatigue Syndrome (CFS), and Chronic Fatigue Immune Deficiency Syndrome (CFIDS) (e.g., [6,7]).

What is clear is that the set of symptoms, although variable in presentation and expressed with different severity in differing patients, is remarkably similar. One symptom seems particularly unique to the disease; post-exertional malaise (PEM) is now considered the “hallmark” symptom [8].

Based upon the belief that this unique set of symptoms should be attributable to a single, unique organism or trigger, considerable effort—spanning at least 40 years—has been spent attempting to identify this causative agent or trigger [9]. The failure to identify a unique, causative agent, coupled with the failure to find any abnormal, routine, clinical laboratory test result, has led many healthcare professionals to conclude that the disease lacks a pathophysiological basis and, therefore, has a psychosomatic etiology. The belief in a psychosomatic origin was applied to both cluster outbreaks and sporadic occurrences of the disease. A retrospective look at one cluster outbreak, which in fact was the occurrence of several cluster outbreaks at several locations of the Royal Free Hospital in London, led to the hypothesis of the disease being mass hysteria [10]. More recently, the mass hysteria hypothesis was challenged and discredited [11].

Latterly, with the advent of social media, patients have been able to self-identify, organize into groups, and advocate for more research and an increase in the number and effectiveness of symptom relief protocols. Their awareness of diseases on both sides of the Atlantic, with almost identical sets of symptoms, has led to the realization that the ME described in the UK and the CFS described in the US are sufficiently concordant in presentation and time-course to be considered overlapping conditions. In 2011, the US National Institutes of Health concluded its CFS State of Knowledge Workshop by announcing the amalgamation of the two names into CFS/ME [12]. This was also the formulation adopted by the Chief Medical Officer’s Working Group in the UK in 2002, when it concluded that the illness was a genuine clinical entity [13]. Afterwards, patient advocates lobbied for the more pathological-sounding name to be placed first. The name ME/CFS was created. The name ME/CFS is currently used despite the 2015 recommendation of the US IOM (Institute of Medicine subsequently renamed the National Academy of Medicine) to have the disease characterized by its cardinal feature and be called Systemic Exertion Intolerance Disease (SEID) [5].

The IOM report of 2015 also declared ME/CFS a disease, as opposed to its classification of being a syndrome [14], based upon the severity of the illness and its unique set of symptoms [5]. Nevertheless, and important for the hypothesis put forward here, ME/CFS remains technically a syndrome: a collection of symptoms of unknown etiology. Much work directed towards identifying the underlying pathology has been undertaken across the world, in many locations including North America and Europe, where the European ME/CFS Research Network (EUROMENE), established in 2006, has, with funding from the European Union’s COST (Cooperation in Science and Technology) program, helped to address this issue (COST project CA15111) [15].

The theme of this issue of Medicina was conceived before the onset of the COVID-19 pandemic. It focuses on the causes of ME/CFS, its clinical features, and its diagnosis, but we cannot ignore the reality that we are at a moment where a pandemic virus is compelling a new understanding of the etiology of ME/CFS: The symptoms manifested by Longhaul COVID-19 patients conform closely to those manifested by ME/CFS patients, and there is some evidence that similar pathological processes, including oxidative stress and mitochondrial dysfunction, are involved in both [16]. This strongly argues against ME/CFS being caused by an unknown trigger. More likely, SARS-CoV-2 will replace the Epstein-Barr virus as being the most frequent precipitating event for ME/CFS or Longhaul COVID-19. While Epstein-Barr virus may have previously been the most frequent precipitant of ME/CFS, other viruses have been reported [17,18]. Little attention has been paid to these reports in an effort to identify a unique causation organism for ME/CFS. However, if science and discipline are to prevail, the explanation of the etiology of ME/CFS must include all identified precipitating events. Such an explanation, inclusive of all viral triggers, has not been put forward up to this time, and other triggers could not be excluded (e.g., Ehlers Danlos Syndrome, cervical spine compression, post-traumatic injury, toxic exposure, or a metabolic defect) [19]. We now put forward such a unifying hypothesis.

In consideration of the appearance of ME/CFS subsequent to the acute infection or to the reactivation of chronic/persistent infection of multiple viral species, coupled with the undeniable appearance of similar symptoms subsequent to the acute phase of SARS-CoV-2, we suggest that the set of symptoms known as ME/CFS and Longhaul COVID-19 should be described as the Post Active Phase of Infection Syndromes (PAPIS). The reason why some viruses are capable of producing PAPIS and capable of doing so more severely than others is unknown. Why some patients acquire PAPIS while others do not is also unknown, however, knowing that PAPIS exists, and that the number of patients exhibiting them will dramatically increase during and subsequent to the COVID-19 pandemic, we need to explore the pathophysiological mechanisms underlying PAPIS. The similarities of symptoms between PAPIS triggered by different viral infections suggest that many of the underlying biochemical pathways and pathophysiological mechanisms will be similar, and perhaps the same. Elucidating these pathways should suggest more effective treatments, if not cures, for these symptoms. ME/CFS patients have suffered far too long. Longhaul COVID-19 patients should not experience a similar fate, and will be far too numerous to be ignored or relegated to the unemployable disabled.

Although presently unknown, the pathophysiological basis of ME/CFS and Longhaul COVID-19 symptoms should no longer be denied. Within EUROMENE, expert consensus has been developed on the diagnosis, service provision, and care of people with ME/CFS in Europe [20], and this is by no means overdue, as, without such consensus, the socioeconomic impact on the whole of the rest of society will, in the aftermath of the COVID-19 pandemic, be immense [21]. Researchers and clinicians need to admit: (1) It is not possible to find what does not exist, (2) Treatments will fail when they do not correct the underlying pathophysiology, and (3) Careful observation and correlation will yield clues to the biochemical and pathophysiological mechanisms underlying these chronic symptoms. For many of its symptoms, Longhaul COVID-19 is not like ME/CFS; it is ME/CFS. While many Longhaul COVID-19 patients will satisfy one or more of the case definitions of ME/CFS, it must be recognized that Longhaul COVID-19, for many, contains symptoms that are other than ME/CFS. Thus, while there is an overlap of the two syndromes, they cannot be considered synonymous. Nevertheless, a focus on PAPIS research is likely to lead to therapies that will make both ME/CFS and Longhaul COVID-19 patients well again.

## Data Availability

Not applicable.
